# Parity, age at first birth, and risk of death from brain cancer: a population-based cohort study in Taiwan

**DOI:** 10.1186/1471-2458-12-857

**Published:** 2012-10-09

**Authors:** Hui-Fen Chiu, Chih-Cheng Chen, Shang-Shyue Tsai, Shu-Chen Ho, Chun-Yuh Yang

**Affiliations:** 1 Department of Pharmacology, College of Medicine, Kaohsiung Medical University, Kaohsiung, Taiwan; 2 Department of Pediatrics, Chang-Gung Memorial Hospital, Kaohsiung Medical Center, Chang-Gung University, College of Medicine, Kaohsiung, Taiwan; 3 Department of Healthcare Administration, I-Shou University, Kaohsiung, Taiwan; 4 Department of Public Health, College of Health Sciences, Kaohsiung Medical University, Kaohsiung, Taiwan

## Abstract

**Background:**

This study was undertaken to examine whether there is an association between parity and age at first birth and risk of death from brain cancer.

**Methods:**

The study cohort consisted of 1,292,462 women who had a first and singleton childbirth between Jan. 1, 1978 and Dec. 31, 1987. We tracked each woman from the time of their first childbirth to December 31, 2009, and their vital status was ascertained by linking records with the computerized mortality database. Cox proportional hazard regression models were used to estimate the hazard ratios (HR) of death from brain cancer associated with parity and age at first birth.

**Results:**

There were 316 brain cancer deaths during 34,980,246 person-years of follow-up. The mortality rate of brain cancer was 0.90 cases per 100,000 person-years. The adjusted HR was 1.35 (95% CI= 0.91-2.01) for women who gave birth between 21 and 25, 1.61 (95% CI=1.05-2.45) for women who gave birth after 25 years of age, respectively, when compared with women who gave birth less than 20 years. A trend of increasing risk of brain cancer was seen with increasing age at first birth. The adjusted HR were 0.73 (95% CI= 0.53-0.99) for women who had 2 children, and 0.60 (95% CI =0.43-0.83) for women with 3 or more births, respectively, when compared with women who had given birth to only 1 child. There was a significant decreasing trend in the HRs of brain cancer with increasing parity.

**Conclusions:**

This study provides evidence that reproductive factors (parity and early age at first birth) may confer a protective effect on the risk of death from brain cancer.

## Background

In Taiwan, brain cancer is the 15th leading cause of cancer mortality for males and the 13th for females [1]. The age-adjusted mortality rate for brain cancer was 2.0 per 100,000 among males and 1.4 among females in 2009. There is substantial geographic variation in brain cancer mortality within the country.

Little is known regarding the etiology of brain cancer. Ionizing radiation is the most consistently accepted causal risk factors for brain cancer [2], and this explains only a small fraction of cases [3], leaving the majority of cases with unknown causes [4].

The incidence rate of glioma is about 1.5 times greater in men than in women [5,6]. The higher incidence in men becomes evident around the age of female menarche, reaching a maximum around the age of menopause and diminishing thereafter [6], suggesting a possible protective effects of female hormones on the development of brain cancer [6]. The influence of sex hormones on brain cancer risk is supported by the presence of steroid hormone receptors in both normal and malignant glial cells [7-9]. Furthermore, studies of transplanted glioblastoma cell lines in mice and rats have found significantly faster tumor growth rate and poor survival in males [10,11].

The role of parity and age at first birth in the etiology of brain cancer in women have received only limited attention in the literature, and the results have been inconsistent. Some studies reported inverse associations between increasing parity and the risk of brain cancer [12-14] and others reported no association [4,15-24]. Likewise, reported associations between younger age at first birth and brain cancer risk have been inconsistent, with one study reporting decreased risk [17] and others reporting no association [4,13,14,16,18-22].

To date, there are limited data regarding the relationship between reproductive factors and brain cancer. The above-mentioned epidemiologic studies have been conducted on high-incidence populations in Europe and North America and study results have not been consistent. The present study was carried out because no studies have been conducted on low-incidence populations such as Asians. We studied a cohort of women who experienced a first and singleton childbirth between January 1, 1978 and December 31, 1987 to explore further the association between parity, age at first birth and the risk of brain cancer in Taiwan.

## Methods

### Data source

Registration of births is required by law in Taiwan. It is the responsibility of the parents or the family to register infant births at a local household registration office within 15 days. The Birth Registration System, which is managed by the Department of Interior, released computerized data on live births since 1978. The registration form, which requests information on maternal age, education, parity, gestational age, date of delivery, infant gender, and birth weight, is completed by the physician attending the delivery. Because most deliveries in Taiwan take place in either a hospital or clinic [25] and the birth certificates are completed by physicians attending the delivery and it is mandatory to register all live births at local household registration offices, the birth registration data are considered complete, reliable and accurate. These data have been used in our previous studies [26,27].

### Study population

The study cohort consisted of all women with a record of a first and singleton childbirth in the Birth Register between January 1, 1978 and December 31, 1987. There were 1,333,312 first and singleton births occurred in Taiwan between 1978–1987. Information on any subsequent births was also retrieved from the Birth Register. Of the 1,399,312 primiparous women, 106,850 subjects were excluded because data were missing on at least one variable such as maternal age (n=100,099), years of schooling (n=382), marital status (n=2665), or birth place (n=4535). These exclusion left 1,292,462 women with complete information for the analysis. Their details have already been described in an earlier publication [27].

### Follow-up

Each woman has her own unique personal identification number. Using this number, we tracked each woman from the time of their first childbirth to December 31, 2009, and their vital status was ascertained by linking records with the computerized mortality database, identifying the date of any deaths occurring in this cohort. Of the 1,292,462 women followed, none had a missing personal identification number; therefore, all cohort members were followed up. Since it is mandatory to register death certificates at local household registration offices, the mortality statistics in Taiwan were considered to be highly accurate and complete [28].

### Statistics

The person-years of follow-up for each woman was calculated from the date of first childbirth to the date of death or December 31, 2009. Death rates were calculated by dividing the number of deaths from brain cancer by the number of person-years of follow-up. Cox proportional hazard regression models were used to estimate the hazard ratios (HRs) of death from brain cancer associated with parity (the number of children recorded in the last childbirth record of each woman registered during follow-up) and age at first childbirth. The 95% confidence intervals (CIs) for the RRs were also calculated. Brain cancer is defined according to the International Classification of Disease, Injury, and Causes of Death (9th revision) (ICD code 191). It includes malignant neoplasm of the cerebrum, except lobes and ventricles (191.0); malignant neoplasm of the frontal lobe (191.1); malignant neoplasm of the temporal lobe (191.2); malignant neoplasm of the parietal lobe (191.3); malignant neoplasm of the occipital lobe (191.4); malignant neoplasm of the ventricle (191.5); malignant neoplasm of the cerebellum (191.6); malignant neoplasm of the brain stem (191.7); malignant neoplasm of other parts of brain (191.8); and malignant neoplasm of brain, unspecified (191.9). The variables in the final model included age at first childbirth (≤ 20, 21–25, > 25), parity (1, 2, 3 or more), marital status (married, unmarried), years of schooling (≤9, > 9 years), and birth place (hospital/clinic, home/other). The proportional hazards assumption was assessed for all above-mentioned variables, and no violations were observed. Analyses were performed using the SAS statistical package (version 9.2, SAS Institute Inc). All statistical tests were two-sided; p values of less than < 0.05 were considered to be statistically significant.

## Results

Altogether 1,292,462 primiparous women with complete information were included in the analysis. A total of 34,980,246 person-years were observed during the follow-up period from the time of their first childbirth to December 31, 2009. There were 316 brain cancer deaths, yielding a mortality rate of 0.90 cases per 100,000 person-years.

Table 1 gives the numbers of person-years of follow-up and brain cancer deaths by age at recruitment (age at first birth), parity, marital status, years of schooling, and birth place. The mortality rate was 1.32 among women who had given birth to one child, 0.94 among those who had had two children, and 0.76 among those who had given birth to 3 or more children.

**Table 1:** Demographic characteristics of the study cohort

**Variables**	**No. of subjects**	**Follow-up person-years**	**No. of death from brain cancer**	**Mortality Rate (per 100,000 person-years)**
Age at recruitment (1^st^ birth)				
≤ 20	158,292	4,544,456.00	30	0.66
21-25	701,650	19,032,158.50	164	0.86
> 25	432,520	11,403,631.50	122	1.07
Parity				
1	157,207	4,170,772.33	55	1.32
2	564,727	15,124,112.33	142	0.94
3+	570,528	15,685,361.33	119	0.76
Marital status				
Married	1,260,615	34,115,479.25	309	0.91
Not married	31,847	864,766.75	7	0.81
Years of schooling				
≤ 9y	722,518	19,850,938.17	176	0.89
> 9y	569,944	15,129,307.83	140	0.93
Birth place				
Hospital / clinic	1,245,925	33,638,862.83	303	0.90
Home / other	46,537	1,341,383.17	13	0.97

The multivariate-adjusted HR and 95% CIs are shown in Table 2. An older age at first birth was associated with an increased brain cancer risk. The adjusted HR was 1.35 (95% CI= 0.91-2.01) for women who gave birth between 21 and 25 years, 1.61 (95% CI=1.05-2.45) for women who gave birth after 25 years, respectively, when compared with women who gave birth younger than 20 years. A trend of increasing risk of brain cancer was seen with increasing age at first birth (p for trend=0.024).

**Table 2:** Association between parity, age at first birth, and relative risk of death from brain cancer over a 32-year follow-up period

**Variables**	**Crude HR (95%CI)**	Multivariate-Adjusted HR^*^(95% CI)
Age at recruitment (1^st^ birth)		
≤ 20	1.00	1.00
21-25	1.36 (0.92~2.01)	1.35 (0.91~2.01)
> 25	1.73 (1.16~2.58)	1.61 (1.05~2.45)
	*p=0.0034 for linear trend*	*p=0.0238 for linear trend*
Parity		
1	1.00	1.00
2	0.71 (0.52~0.97)	0.73 (0.53~0.99)
3+	0.56 (0.41~0.78)	0.60 (0.43~0.83)
	*p=0.0005 for linear trend*	*p =0.0034 for linear trend*
Marital status		
Married	1.00	1.00
Not married	0.89 (0.42~1.88)	0.81 (0.38~1.73)
Years of schooling		
≤ 9 y	1.00	1.00
> 9y	1.07 (0.86~1.34)	0.93 (0.73~1.18)
Birth place		
Hospital / clinic	1.00	1.00
Home / other	1.03 (0.59~1.80)	1.16 (0.66~2.03)

^*^ mutually adjusted.

After adjustment for age at first birth, marital status, years of schooling, and birth place, the adjusted HR were 0.73 (95% CI= 0.53-0.99) for women who had 2 children, and 0.60 (95% CI =0.43-0.83) for women with 3 or more births, respectively, when compared with women who had given birth to only 1 child. There was a statistically significant decreasing trend in the adjusted HRs for brain cancer with increasing parity (p for trend=0.003).

## Discussion

To our knowledge, this is the largest cohort (n=1,292,462 women) published to date to examine the relationship between reproductive factors (parity and age at first birth) and brain cancer risk. In this prospective cohort study, we found a statistically significant decreased risk of brain cancer with increasing parity. Our findings of a reduced risk of brain cancer associated with higher parity is in agreement with previous studies [12-14], but is not in agreement with other studies that reported no association with parity [4,15-24].

The mechanism by which increased parity may confer protection against the future development of brain cancer in women remains unclear. Pregnancy elevates serum estrogen levels approximately 100-fold [29]. Increasing parity is associated with an overall increase in lifetime estrogen exposure. There is experimental evidence that estrogen inhibits proliferation of glioma [30,31] and induces cell death [30,32]. In addition, estrogen has been shown to reduce glutamate toxicity in glial cells [33] and speeds up the process of repair after brain trauma [34]. Thus, if estrogens are associated with a reduced risk of brain cancer, we would expect pregnancy to offer some protection from brain cancer. Our data provide support for this hypothesis. However, it is unknown whether estrogen or other hormones play a role in the development of brain cancer. Support for a possible role of sex hormones in gliogenesis comes from the fact that some gliomas express estrogen receptors [7,9]. Furthermore, aromatase, an enzyme with high levels in human and rat glioblastomas, can convert testosterone to estradiol [35].

In the present prospective cohort study, we found that risk of brain cancer increased with increasing age at first birth after adjusting for parity. Our finding of a positive association between age at first birth and brain cancer risk agrees with one previous study [17] but not with other studies that reported no association with age at first birth [4,13,14,16,18-24]. The reasons are unknown. It may be that a younger age at first birth played a protective role in brain cancer risk via elevated levels of some hormones (including estrogen and progesterone) during pregnancy. Moreover, it has been found that age at menarche (and thus exposure to period estrogens stimulation) is related to age at which a women delivers her first child [36]. Earlier exposure to regular menstrual cycles and the concomitant earlier increase in exposure to estrogens may protect against tumor initiation during this early life stage [17]. Our finding of an increased risk of brain cancer associated with older age at first birth is in keeping with the hypothesis that estrogen exposure is protective with respect to brain cancer risk. However, because there is only limited evidence to date for a positive association between age at first birth and risk of death from brain cancer, the possibility that this is a chance finding also needs to be considered. Clearly, more work will be needed before the influence of age at first birth on the risk of brain cancer is understood.

To our knowledge, ours is the first prospective cohort study to report a protective effect of parity and early age at first birth on the subsequent risk of death from brain cancer. However, these results are inconsistent with most studies published to date. The reasons for these differences in findings are unknown. Only three prospective studies have examined the relationship between parity and age at first birth and brain cancer risk [18,19,22]. Most of previous studies used case–control design [4,12-17,20,21,23,24]. The main strength of this investigation is its prospective study design, which eliminates the possibility of recall bias. Also, the complete population coverage and follow-up made possible by the national identification number has left the study without selection bias. The mean age at baseline was 24.33 years in this study. Women included in our study tended to be younger than those in previous cohort studies (ranging from 48.5 to 55.9 [18,19,22]. In addition, the number and type of potential confounders taken into account varied between studies. The relative risk estimate may therefore be different between studies [37]. Also, information on exogenous hormone use (the use of oral contraceptives (OC) and hormone replacement therapy (HRT)) and cumulative number of menstrual cycles (age at menarch and menopause) were not available in this study. Previous prospective cohort studies have included all different exposures in a multivariate analysis. This analysis could help to better assess effect of each hormonal factor. Finally, age at first birth may be different in different countries and during different periods. The mean age at first birth was 24.33 in our cohort. This value was increased during the study period (ranged from 23.3 to 28.1 in 2006). Likewise, same temporal differences may exist for European countries.

Mortality data have been widely used to generate epidemiologic hypotheses, despite their inherent limitations. The completeness and accuracy of the death registration system should be evaluated before any conclusion based on the mortality analysis is made. In the event of a death in Taiwan, the decedent’s family is required to obtain a death certificate from the hospital or local community clinic, which then must be submitted to the household registration office in order to cancel the decedent’s household registration. The death certificate is required in order to have the decedent’s body buried or cremated. Death certificates must be completed by physicians in Taiwan. It is also mandatory to register all deaths at local household registration offices, the death registration is accurate, reliable and complete.

Taiwan is a small island with a convenient communication network. It is believed that all brain cancer cases had access to medical care. Mortality data rather than data on inpatient cases was used to assess the association between parity, age at first birth, and brain cancer in this study. The mortality of a disease is a function of its incidence and fatality. The 5-year survival rate for brain cancer has been reported to be as low as 30% in United States [2]. Deaths from brain cancer may therefore be regarded as a reasonable indicator of the incidence of brain cancer.

Several studies have found that the use of OCs was associated with a reduced risk of brain cancer [15,17,21]. Other studies, however, have failed to find statistically significant association between the use of OCs and brain cancer [3,4,16,18,19,22]. Regarding HRT, previous studies have largely reported no association between HRT and brain cancer risk [3,4,16-18,22]. We were unable to adjust for these two hormonal factors in the current study due to the lack of available data. Since the use of OC and HRT are low in Taiwan compared with Western countries [38,39], the confounding effect resulting from these two factors should be small, if any exists at all. Furthermore, if the association between these two potential confounding variables and the risk of brain cancer is not as strong as the one that has been observed for parity and age at first birth, adjustment of these variables will not qualitatively change the conclusion.

Smoking was associated with the incidence of brain tumors [40-42]. There is unfortunately no information available on individual smoking habits and thus it could not be adjusted in the analysis. The smoking prevalence for females was only about 4.2% in Taiwan [43]. We think that the degree to which not controlling for this variable may have affected our results should be small if it existed at all. Nevertheless, the problem of possible confounding from smoking should be evaluated. Smoking habits account for at least part of the socioeconomic differentials, with rates of smoking considerably higher among those with lower levels of education. In this study, years of schooling was used as a proxy for socioeconomic status and was included as a control variable in the multivariate analysis. We therefore may have partially indirectly adjusted for the confounding effect resulting from smoking.

Ionizing radiation, occupational exposure to solvents, and electromagnetic fields are the most important risk factors for brain cancer [2]. There is no information available on these variables for individual study subjects and thus they could not be adjusted for in the analysis. However, there is no reason to believe that there would be any correlation between these variables and parity and age at first birth.

Some potential limitations of this study needed to be noted. First, our computerized death-certificate database does not specify the tumor type and the histological classification for the brain cancer cases. We therefore could not estimate the risk of brain cancer in more detail in relation to risk for specific brain cancer subtypes. Second, data on the accuracy of brain cancer diagnosis are not available in Taiwan and misclassification is possible. However, this misclassification is likely to be nondifferential (i.e., unlikely to be related parity) and therefore would tend to underestimate rather overestimate the true association. Third, Taiwan’s vital records and birth registration system covers only live births and exclude stillbirths and abortions. We were therefore unable to examine the possible role of gravidity on the brain cancer risk. Fourth, by design, our study focused solely on mortality among parous women. We were unable to examine the possible role of nulliparity on the risk of brain cancer. The generalizability of our findings is thus limited. Fifth, although the number of person-years are large, the number of of brain cancer deaths is relatively small (n=316). The power of this study was therefore relatively limited. The possibility that this is a chance finding also needs to be considered.

## Conclusion

We found that there was a trend for increasing parity to be associated with decreasing risk for brain cancer. In addition, we found that risk for brain cancer increased with increasing age at first birth. This study provides evidence that reproductive factors (parity and early age at first birth) may confer a protective effect on the risk of brain cancer.

## Competing interests

The authors declare that they have no competing interests.

## Authors’ contributions

HFC wrote the manuscript. CCC and SST provided essential insight into the interpretation of the results. SCH did the statistical analysis. CYY contributed to study design and interpretation of the data. He had full access to all of the data in the study and took responsibility for the integrity of the data and the accuracy of the data analysis. All authors read and approved the final manuscript.

## Pre-publication history

The pre-publication history for this paper can be accessed here:

http://www.biomedcentral.com/1471-2458/12/857/prepub
